# Challenges in Management of VTE in Children With Cancer: Risk Factors and Treatment Options

**DOI:** 10.3389/fped.2022.855162

**Published:** 2022-04-07

**Authors:** Nasrin Samji, Mihir D. Bhatt, Ketan Kulkarni

**Affiliations:** ^1^Department of Pediatrics, Division of Hematology Oncology, McMaster University, Hamilton, ON, Canada; ^2^Department of Pediatrics, Division of Hematology Oncology, Dalhousie University and Izaak Walton Killam (IWK) Health Centre, Halifax, NS, Canada

**Keywords:** Pediatric Thrombosis, cancer associated thrombosis, childhood VTE, direct-acting oral anticoagulant (DOAC), malignancy and thrombosis

## Abstract

Venous thromboembolism (VTE) occurs in 2.1 to up to 50% of children with cancer and contributes to long term morbidity as well as early mortality in this population. Pediatric patients with malignancy are predisposed to VTE due to the prothrombotic nature of cancer and its associated coagulopathies as well as chemotherapeutic agents, use of central venous catheters, surgery, radiotherapy, and concomitant thrombophilia. Management of thrombosis in this population is challenging due to concomitant thrombocytopenia, associated bleeding risks, concurrent co-morbidities, and toxicities of therapy. The aim of this paper is to highlight clinically relevant issues and management dilemmas using clinical vignettes. We review the clinical significance of asymptomatic and symptomatic thrombosis, examine the various options for asparaginase-associated thrombosis, address the role and controversies of direct oral anticoagulants, and describe our approach to managing anticoagulation therapy in the context of chemotherapy-induced thrombocytopenia.

## Introduction

Thrombotic complications are well-recognized in children with cancer and can lead to significant morbidity (1–3) and reduced survival (4–6). The incidence greatly exceeds that of the general pediatric population with an incidence of 2.1–16% for symptomatic thrombosis and up to 50% when including asymptomatic events (7–10). The reported cumulative incidence of life-threatening venous thromboembolism (VTE) is ~0.36% (8, 11). Clinically significant or life-threatening thrombosis in this fragile population may lead to acute interventions, undue stress on families, and increased resource utilization such as more and lengthier hospitalizations, additional use of radiologic resources, and multidisciplinary expertise from thrombosis, vascular surgeons, intensive care unit teams, as well as several pediatric subspecialists. VTE can also affect the trajectory of oncologic treatment and may lead to delayed surgery and modifications in the therapy plan, which can impact the overall outcome (6, 12).

The pathophysiology of cancer associated thrombosis (CAT) is not fully understood, however, cancer can directly affect the components of Virchow's triad. Cancer is thought to induce a prothrombotic state through direct activation of platelets and endothelial damage. Additionally, exposure of tissue factor from the subendothelial layer as well as its presence on circulating tumor microparticles, may also play a significant role in development of thrombosis (13). Studies have reported alterations in the hemostatic balance of children with cancer, such as elevation of von Willebrand Factor antigen, factor VIII, and increased thrombin generation. Reductions in natural anticoagulants such as protein C has also been documented in patients with acute lymphoblastic leukemia (14). Mass effect from solid tumors causing stasis of blood flow may also contribute to thrombosis in this population (15).

**Figure 1:**
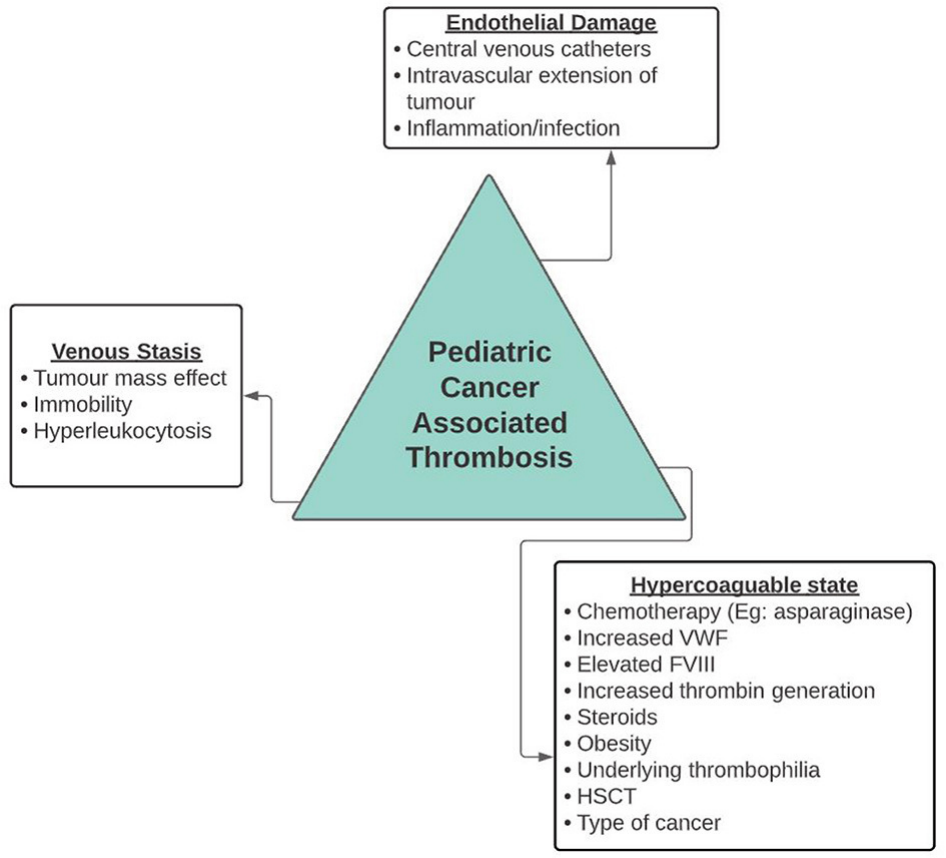
Risk factors for cancer associated thrombosis.

The etiology and risk factors contributing to development of VTE in children with cancer is thought to be multifactorial, contingent on the type of cancer, its burden, and location (Figure 1) (8, 11). In a population cohort study in Canada, conducted by Pelland-Marcotte et al. (8), patients with leukemia were found to have the highest incidence of symptomatic thrombosis (5.6%), followed by lymphoma (4.4%), extracranial tumors (3.7%), and brain tumors (1.0%). Other risk factors documented in the literature include patient related factors such as older age, BMI, blood type O group, and underlying thrombophilia as well as treatment related risk factors such as chemotherapeutic agents and central venous lines (CVLs) (8, 11). Therapy implicated in cancer include asparaginase in patients with hematologic malignancies and platinum agents in patients with solid tumors; anthracyclines have been associated with both populations (8).

Most children with cancer who develop symptomatic thrombosis receive medical intervention (8), however, there are a variety of challenges that can occur in this population due to concurrent morbidities secondary to malignancy or toxicities of therapy. Children with cancer acquire an increased risk of bleeding because of chemotherapy or malignancy induced thrombocytopenia, hypervascularization of tumors, proximity of tumors to vascular structures, hyperleukocytosis, coagulopathy associated with malignancy, infections, chemotherapy, typhlitis, and vitamin K deficiency from poor nutritional status. The use of anticoagulants and management of bleeding risk should be addressed in every child. Additionally, the paucity of guidelines for prevention and treatment of childhood associated thrombosis can lead to lack of clarity in terms of management and variability in approach to treatment.

This paper aims to highlight some key clinically relevant issues in pediatric cancer associated thrombosis (CAT) and management dilemmas using clinical vignettes with some practical recommendations that can be considered in your approach to treatment despite the lack of randomized clinical trials to support treatment plans.

## Clinical Case 1

A 13-year-old female diagnosed with Ewing Sarcoma develops a right femoral DVT associated with post-operative immobility. She has just received a cycle of chemotherapy and her currently platelet count is 175. The patient is needle averse and asks if there is an oral alternative.

The primary issues we will address for this case include the following: (1) what oral anticoagulation options are available? (2) who would be eligible for DOACs? (3) what is the optimal duration of treatment, and (4) does this patient require long term anticoagulation?

Traditionally, unfractionated heparin (UFH) and low molecular weight heparin (LMWH) have been the standard of care in pediatric oncology patients, in the absence of contraindications. Oral anticoagulants such as warfarin were typically avoided due multiple food and drug interactions (16). However, with the evolution newer direct oral anticoagulants (DOACs) and the recent clinical trial outcomes, oral therapeutic anticoagulation options are expanding rapidly and will continue to evolve (17–20). DOACs may overcome some limitations of standard anticoagulation such as needle phobias and routine monitoring (16). After undergoing phase 3 clinical trials, Rivaroxaban, a direct factor Xa inhibitor and Dabigatran, a direct thrombin inhibitor, are the only DOACs approved in pediatrics in Canada and the USA, respectively. Alternate DOACs are currently under investigation through the Edoxaban Hokusai VTE PEDIATRICS study, which is a phase 3 clinical trial, and as part of the Children's Oncology Group ACCL1333 study, whereby apixaban is being trialed as prophylactic anticoagulation for induction therapy in patients with acute lymphoblastic leukemia (ALL) (21).

The EINSTEIN-Jr and DIVERSITY clinical trials were sizable studies that showed non-inferiority of rivaroxaban and dabigatran, respectively, to the standard of care antithrombotic medications (UFH and LWMH) in the pediatric population (20, 22). The EINSTEIN-Jr trial studied 520 pediatric patients, 335 who were randomized to the rivaroxaban arm; 50% of the population had non-catheter, non-CNS related venous thromboembolisms while 25% had CVL-associated VTEs. Rivaroxaban showed significant improvement in clot resolution on repeat imaging (*p* = 0.01), low risk of recurrence of thrombosis and similar rates of clinically relevant bleeding, compared to the standard of care (23). The DIVERSITY trial enrolled 328 children, 66% of whom were assigned to the Dabigatran group. Thrombus resolution and recurrence were similar in the dabigatran and standard of care groups. Bleeding events were similar in both groups with 24 vs. 22% in the standard of care vs. Dabigatran groups, respectively (18).

Both clinical trials examined DOAC safety and efficacy in a diversity of patients, some of whom had oncologic diagnoses. Of the 520 children in the EINSTEIN-JR trial, 10–12% had oncologic diagnoses; 25 patients with malignant hematologic disease and 15 patients with solid tumors received rivaroxaban therapy (23, 24). Eleven percent of the population in the DIVERSITY trial had either an active or past medical history of cancer (18). The above data does suggest that DOACs are potentially efficacious for the treatment of pediatric CAT. However, it should be noted that further sub-group analysis within the oncology population was not completed and the number of cancer patients within these studies was small. Additionally, the DIVERSITY trial also excluded patients who received asparaginase and peg-asparaginase within 1 and 2 weeks, respectively, of administration (18).

Both dabigatran and rivaroxaban offers bodyweight-adjusted anticoagulation treatments with usable formulations for the pediatric population. At present there is no data to support the up-front use of DOACs at diagnosis as both EINSTEIN-Jr and DIVERSITY trials started the DOAC after a minimum of 5 days of standard of care anticoagulant with either UFH or LMWH (17, 18, 20). Additionally, while DOACs show promise in the pediatric population, co-morbidities associated with cancer can impact the ability to absorb, metabolize, or excrete the drug effectively. This may limit their use in this population and result in careful selection of oncology patients for eligibility for use (Table 1).

**Table 1:** Considerations for DOAC use.

**Considerations prior to DOAC use in pediatric oncology patients**
•Frequency of interventions (e.g., Lumbar puncture)
•Bleeding risk
•Anticipated duration of thrombocytopenia/anemia
•Patient preference
•Availability of appropriate oral suspension/solution
•Proximity to asparaginase use
•Location of thrombus (e.g., CSVT)
•Concomitant use of strong CYP P450 and P-glycoprotein inducers or inhibitors
•Anorexia/intolerance to oral medications
•Severe chemotherapy induced nausea and vomiting

Individuals with cancer may endure severe chemotherapy induced nausea and vomiting as well as anorexia leading to impaired absorption of oral medication and possible limitations in bioavailability of the medication. Additionally, children with cancer may have concurrent renal or hepatic insufficiencies secondary to location of the primary tumor, metastasis, or due to toxicities of therapy. Lastly, various anti-cancer agents as well as supportive care agents can significantly affect the cytochrome P450 and P-glycoprotein pathways which are used in their metabolism (25).

### How We Would Treat This Patient

This patient had a symptomatic DVT secondary to immobilization with an underlying cancer disorder. Precluding any contraindications to anticoagulation, we would start with LMWH as there is no evidence available that supports up front DOAC use. Like the EINSTEIN-Jr and DIVERSITY trials, it would be prudent to check a CBC, coagulation studies, renal function tests, bilirubin, and transaminases as we would avoid DOAC use in patients with an estimated GFR of <30 mL/min/1.73 m^2^, hepatic disease (ALT 5 × upper limit of normal; bilirubin > 2 × the upper limit of normal), thrombocytopenia (platelet count <50,000), and coagulopathy (18, 24). Additionally, we would check for use of strong inducers and inhibitors of cytochrome P450 isoenzyme 3A4 and P-glycoprotein such as voriconazole and posaconazole. If the child's laboratory values are normal and there are no other contraindications to use, we would suggest starting a weight adjusted DOAC dose at least 5–10 days after initiation of anticoagulation. No anticoagulation monitoring would be necessary thereafter. We would advise clinical assessment of bleeding and adherence at every clinic visit by the primary oncologist as well as routine monitoring of liver and renal function tests monthly for the first 3 months of therapy. Additionally, given the variance in weight a child can have during chemotherapy, it would be prudent to document a new weight monthly to ensure no dose adjustments are necessary.

Given that this thrombus was a non-catheter related DVT that was provoked, we would recommend at minimum 3 months of treatment or longer should the risk factor of immobility remain. The efficacy and safety of Rivaroxaban beyond 3 months has not been studied in the pediatric population. However, a single arm phase 3 trial assessing the safety of dabigatran for secondary prevention of VTE was conducted whereby patients were treated with dabigatran for up to 12 months (17). Of the 204 patients that participated in this trial, 5.5% had underlying hematologic malignancy and 2% had a history of solid cancer. Although there was no comparator group, Dabigatran showed similar efficacy and safety to the DIVERSITY study, demonstrating a low frequency of VTE recurrence (1.0%) as well as a low incidence of major bleeding (1.5%) (17). Therefore, if available dabigatran could be an appropriate alternative for secondary prevention or for extended duration anticoagulation therapy up to 12 months. Notably, a metanalysis conducted by Bidlingmaier et al. (26) showed a recurrence risk of 5.2% for pediatric patients on LMWH for secondary prophylaxis. The Kids-DOTT randomized clinical trial assessed the effect of a shorter duration of anticoagulation therapy on recurrence of VTE. Children who achieved partial or complete recanalization at 6 weeks were randomized to discontinue anticoagulation or to continue anticoagulation for a total duration of 3 months. Results showed that the reduced 6-week course of anticoagulation was non-inferior. However, the study had a small number of pediatric patients with cancer and therefore its clinical application within pediatric oncology may be limited (27).

## Clinical Case 2

A 16-year-old male diagnosed with high-risk acute lymphoblastic leukemia (ALL) is in induction chemotherapy using a Children's Oncology Group protocol. He received PEG-asparaginase 1 week prior and presented to the hospital with 2 days of headache and 1 day of blurry vision. An MRI/V of the head is completed and showed a superior sagittal vein thrombosis with extension to the cortical veins. The primary issues we will address for this case are: (1) anticoagulation options, (2) duration of therapy, (3) what to do with asparaginase post-cerebrosinus venous thrombosis (CSVT), and (4) what about primary prophylaxis?

Thrombosis occurs in up to 36% of pediatric patients with ALL, many of which occur during induction remission (28) with a median time of 107 days (4). A recent study demonstrated an increased risk among high/very high-risk leukemia patients (8.6%) vs. standard/low-risk patients (3.5%) (4). Patients with ALL are at particular risk for VTE due to multiple risk factors at diagnosis and throughout induction therapy such as endothelial damage from central venous catheter insertion, the inherent prothrombotic state associated with cancer, as well as pro-thrombotic medications that are administered together during induction therapy including asparaginase, corticosteroids, and anthracyclines (8).

Asparaginase is a chemotherapeutic agent that diminishes plasma asparagine, which leukemic cells are dependent on for proliferation (29). Pegylated asparaginase has an extended half-life, prolonging the depletion of serum asparaginase in the blood and cerebrospinal fluid (29). Notably, it creates a hemostatic imbalance as it significantly alters hepatically synthesized plasma proteins, affecting many coagulation factors and inhibitors such as antithrombin III. This hemostatic imbalance can ultimately lead to coagulopathy with hemorrhagic and thrombotic complications (30), often having a predilection for CSVT (28). Thrombotic risk is associated with increased age, due to reduced natural anticoagulants and fibrinolysis. Additionally, longer duration of asparaginase at lower doses has been correlated with an increased risk of thrombosis (28).

Thrombotic complications associated with asparaginase often lead to discontinuation and missed doses. The reported cumulative incidence of discontinuation for any reason in AALL0331 and AALL0232 patients was 12.2 ± 0.5% and 25.3 ± 0.8%, respectively (12). Stroke was the reason for discontinuation in 12 (3%) patients in AALL 0331 and 20 (4.1%) patients in AALL0232, the majority of whom did not receive Erwinia substitution. Most importantly, the study showed that discontinuation and missed doses of asparaginase results in a significantly inferior disease-free survival in this patient population (12). Current data on re-challenging pediatric patients following asparaginase-associated thrombosis on a Dana Farber Cancer Institute (DFCI) protocol resulted in recurrence in 17% of patients while on secondary prophylaxis; the recurrence was usually at the original thrombosis site (31). Alternatively, Qureshi et al. (32) report a VTE incidence of 3.2% in patients with ALL who received treatment based on the UK ALL 2003 study, during a period of asparaginase depletion with no subsequent thrombosis during asparaginase re-challenges; 34 of 59 children received secondary anticoagulant prophylaxis. A single institutional retrospective study in North India reported seven patients with CSVT on a Berlin-Frankfurt-Münster (BFM)-based protocol who tolerated asparaginase rechallenge while concomitantly using LMWH (33).

The role of primary prophylactic anticoagulation for this patient population shows promise with LMWH. The Thrombotect study was a significant prospective, randomized trial that compared LMWH prophylaxis vs. activity adjusted antithrombin vs. low dose IV heparin group as the control. Thromboprophylaxis was started on day 8 and ended on day 33 of induction chemotherapy. Outcomes showed a significantly higher VTE incidence in the control group. This was the first trial to show that prophylactic LMWH or antithrombin intervention can significantly reduce VTE, particularly in children above 6 years of age (34). Additionally, a systematic review conducted by Pelland-Marcotte et al. (35) looked at the safety and efficacy of primary thromboprophylaxis and concluded that enoxaparin was the only agent associated with a statistically significant lower odds of VTE compared with the standard of care. Furthermore, 0.7% of patients had major bleeds in this systematic review with no fatal bleeding. Various other prophylactic regimens have been trialed including low dose warfarin for maintenance of CVL patency, which concluded early due to lack of clear benefit along with the PARKAA trial, which aimed to assess the efficacy and safety of prophylactic antithrombin replacement in kids with acute lymphoblastic leukemia treated with asparaginase (35, 36).

The role of prophylactic anticoagulation remains under investigation. Data from the TropicALL study which is assessing the efficacy and safety of thromboprophylaxis with LMWH during asparaginase therapy is eagerly awaited (37). Additionally, the PREVAPIX-ALL study, which is comparing apixaban to the standard of care for prevention of venous thrombosis in pediatric patients with ALL, will provide further insight to primary prophylaxis with apixaban (38). These advances in care are important as patients with VTE have been shown to have a significantly worse overall survival and event free survival.

### How We Would Treat

Anticoagulation is the mainstay of therapy for individuals with ALL and thrombosis. In this case of CSVT, anticoagulation is recommended. However, this determination is dependent on weighing the risk of bleeding and thrombus propagation. In a study conducted by Moharir et al. (39), 85/160 pediatric patients received anticoagulation for CSVT at diagnosis, 6% of whom had major anticoagulant associated ICH. Of the untreated patients, thrombus propagation occurred quite frequently (31%). While either UFH or LMWH can be utilized in this case, provider preference, presence of associated hemorrhage, resources, expertise, and bleed risk may influence the decision. We would also conduct early re-evaluation for anticoagulation associated hemorrhage with CT or MRI. In patients with difficulty achieving therapeutic targets on anticoagulation, evaluation of antithrombin III levels for determination of heparin resistance may be useful.

For long term anticoagulation, DOACs should be used with caution. Subgroup analysis of the EINSTEIN-Jr trial in the CSVT population showed low risk of clinically relevant bleeding (6.8%) and the DIVERSITY study, although not tested statistically, did not contradict the findings of the larger trial (22, 40). Importantly, both studies had small sample sizes and additional investigations may be required to further guide management. At present, the Thom-PED DOAC registry of the International Pediatric Thrombosis Network is conducting a prospective cohort study to obtain further real-world experience regarding safety and efficacy of DOACs in children, including patients with cancer. Interim analysis of 82 patients, 13% of whom had cancer, showed reduced efficacy and more bleeding than previously observed in published trials (41). Therefore, in this case, we would continue to use LMWH for a minimum of 3 months. Given the poor outcomes associated with discontinuation of asparaginase, the option to re-challenge patients with concomitant use of anticoagulation at treatment dosing in the absence of contraindications can be considered. Duration can be further guided by radiologic and symptomatic resolution.

## Clinical Case 3

Five-year-old male with AML undergoes routine cardiac screening prior to anthracycline chemotherapy. An incidental right subclavian vein non-occlusive thrombosis is diagnosed. His hickman remains in place despite use of tPA flushes in the past for catheter malfunction. Ultrasound reports that this is likely an acute non-occlusive thrombosis, occupying 50% of the vessel. He has had no previous symptoms such as edema, pain, or visible collaterals. He is starting his third cycle of chemotherapy and will receive an allogenic hemotopoietic stem cell transplantation in the near future. The primary issues we will address for this issue are: (1) Is there a role for anticoagulation in asymptomatic thrombosis? (2) How do we manage anticoagulation in the setting of cancer induced thrombocytopenia?

CVLs play a key role in pediatric cancer treatment as they facilitate ease of access for frequent blood draws, transfusions, parenteral nutrition, and administration of chemotherapy. Catheter related thrombosis is a leading cause of VTE in the pediatric oncology population, with an incidence ranging from 2 to 50% (9, 10). Differences in study methodology, diagnostic approach to thrombosis, chemotherapeutic agents, underlying cancer diagnosis, and inclusion of asymptomatic thrombosis may account for the variability in incidence (9). Clinical symptoms of catheter related thrombosis include edema of the affected limb, erythema, pain/tenderness, reduced range of motion, warmth, presence of collaterals, distended veins, skin discoloration, catheter malfunction, or sepsis (9). Risk factors include duration of CVL, type of CVL (PICC vs. implantable port), double lumen (vs. single), younger age at diagnosis, and left sided placement of CVLs (10, 42, 43). Complications of catheter associated thrombosis include catheter malfunction, recurrent surgeries for central line replacement, infection, chronic vascular occlusion, thrombosis recurrence, embolization, and death.

Symptomatic thrombosis is often treated with anticoagulation; however, management of asymptomatic CVL-associated thrombosis remains controversial. Asymptomatic thrombosis often occurs within a week of CVL insertion and demonstrates higher incidence with catheters that remain in place for >28 days (44). A systematic review conducted by Hansen et al. (45), reported 210 asymptomatic thrombosis of 2,318 pediatric cancer patients; these asymptomatic thromboses represented half of all VTE's diagnosed, demonstrating their frequency. Current 2018 guidelines from the American Society of Hematology suggests either anticoagulation or no anticoagulation in children with asymptomatic DVT, leaving the choice up to the provider (46). However, a recent systematic review conducted by Sharathkumar et al. (44), recommends assessment of risk of propagation based on the clinical scenario and individualizing the approach to management. In considering these guidelines, we find it important to weigh the risk of bleeding, appreciate the timing of the clot (if identifiable on imagining), persistence of risk factors, risk of recurrence, and understanding of long-term morbidities.

Current literature shows that catheter related thrombosis can lead to morbidity and possibly reduced survival in this population (1, 3, 47). Polen et al. (1) looked at the prevalence of post thrombotic syndrome (PTS) in a cohort of pediatric cancer survivors after central line removal. They found that approximately one-third of patients had symptoms consistent with PTS, most of which were mild. The most common symptoms were increased limb circumference and pain in this population. 2.7% of patients had physically and functionally significant with PTS, which was associated with history of catheter related DVT, central line occlusion, and multiple CVCs. Additionally, patients with PTS signs and symptoms had lower quality of life scores (1). Kuhle et al. (3) assessed the incidence of PTS in children with ALL who had asymptomatic VTE, but radiologic confirmation of DVT with at least 50% obstruction, and found that 50% had findings compatible with a diagnosis of PTS, indicating that asymptomatic thrombosis may result in mild, and clinically relevant long-term ramifications such as pain, risk of recurrence in future high-risk situations, or loss of future vascular access. In contrast, Albisetti et al. found that 45/114 (39.5%) of children were diagnosed radiologically with port-a-cath related DVT, 5 of whom had symptomatic VTE throughout their therapy course. They documented a lower rate of mild PTS of 5.8% in this population, although confirming that port-a-cath related DVTs, even if symptomatic can lead to PTS.

### How We Would Treat

We are suggesting anticoagulation in this pediatric oncology patient with asymptomatic VTE. While there is evidence that asymptomatic thrombosis can lead to post-thrombotic syndrome, there is no evidence on the efficacy of anticoagulation and therefore it would be remiss to suggest its use for all patients. Our decision to utilize anticoagulation is individualized, and is based on bleeding risk, current prothrombotic state, chronicity of the asymptomatic thrombosis, and persistence of risk factors. This patient has catheter malfunction, which has been associated with PTS and therefore choosing to treat has potential to impact long term morbidity. Additionally, this child is due to receive more intensive therapy with hematopoietic stem cell transplant (HSCT), which alters the hemostatic balance. HSCT encourages a prothrombotic state with increased plasma TAT complexes and decreased levels of natural anticoagulants (48). The intention of starting anticoagulation would be to decrease the risk of clot progression and reduce the potential risk for PTS. While this opinion may not be shared by all, if anticoagulation is not started, we would suggest routine surveillance in this patient for detection of extension.

Management of anticoagulation in this child however may be challenging due to the significant bone marrow suppression caused by AML chemotherapy. At present there are no current guidelines regarding safe thresholds or when to transfuse patients. At our institution, we typically keep platelets above 30,000 by transfusing in the first 2 weeks of anticoagulation treatment. Subsequently, we hold anticoagulation for patients who have platelet counts below 20,000, administer half-dose for a platelet count between 20,000 and 30,000, and full dose for a platelet count above 30,000.

## Summary

Management of VTE in pediatric oncology patients is challenging due to the toxicities of therapy, co-morbidities, and bleeding risks. The three cases presented reflect some of the challenging scenarios that are often encountered in clinical practice, which may not have a straightforward answer. We suggest an individualized approach to management with identification of both bleeding and clotting risk factors, which can aid with decision making. When choosing not to treat, close clinical and radiological surveillance for thrombosis extension is recommended. With the advent of precision medicine, a range of multiomics studies have investigated chemotherapy and treatment related toxicity in oncology patients. Early data from adult studies suggests that there may be a strong link between genetics, microbiome, and risk of thrombosis (49–52). However, studies assessing association of genomics, microbiome and metabolome with thrombosis is pediatric cancer patients are lacking. Future studies are needed that evaluate these associations.

## Author Contributions

NS performed the review and wrote the manuscript. MB and KK assisted in performing the review and editing and writing the manuscript. All authors contributed to the article and approved the submitted version.

## Conflict of Interest

The authors declare that the research was conducted in the absence of any commercial or financial relationships that could be construed as a potential conflict of interest.

## Publisher's Note

All claims expressed in this article are solely those of the authors and do not necessarily represent those of their affiliated organizations, or those of the publisher, the editors and the reviewers. Any product that may be evaluated in this article, or claim that may be made by its manufacturer, is not guaranteed or endorsed by the publisher.
